# Stress Status and Related Characteristics among Urban Residents: A Six-Province Capital Cities Study in China

**DOI:** 10.1371/journal.pone.0030521

**Published:** 2012-01-20

**Authors:** Tingzhong Yang, Ian R. H. Rockett, Qiaohong Lv, Randall R. Cottrell

**Affiliations:** 1 Center for Tobacco Control Research, Zhejiang University School of Medicine, Hangzhou, Zhejiang, China; 2 Injury Control Research Center and Department of Community Medicine, West Virginia University, Morgantown, West Virginia, United States of America; 3 Health Promotion and Education Program, School of Human Services, University of Cincinnati, Cincinnati, Ohio, United States of America; Catholic University of Sacred Heart of Rome, Italy

## Abstract

**Objectives:**

To estimate current prevalence levels of stress, and to identify related characteristics among urban residents in China.

**Design:**

A cross-sectional, multilevel study. Selected through multi-stage quota-sampling, survey participants were 4,735 urban residents aged 15 years and older who resided in one of six selected Chinese capital cities. Data were collected on stress levels and sociodemographic characteristics. Stress was assessed using the Perceived Stress Scale, Chinese version (CPSS). A multilevel variance component model was employed to analyze associations between sociodemographic variables and stress.

**Results:**

The mean stress score for urban residents was 22.34 (SD: 3.22), and 36.8% of those surveyed (95% CI: 33.5–40.2%) were severely stressed (>25). Multilevel regression analysis indicated that residents aged 55 years and older were less stressed than residents under age 25. The most educated and higher income earners had lower stress levels than the least educated and poorest. High levels of stress were apparent among all other occupational groups, relative to managers and clerks, except retirees and operational workers. Residents in the north of China exhibited higher stress levels than counterparts in the south.

**Conclusions:**

This study suggests that higher stress levels are positively associated with social class in China. Our findings could inform health policy, guide prevention strategies, and justify the design and implementation of targeted interventions.

## Introduction

Stress is a pervasive aspect of life. Indeed, a certain amount of stress is generally regarded as stimulating and life enhancing, although high persistent levels can induce serious health problems, including mental illness and physical disease [1], [2]. Since 1978, China has been transitioning from a centralized to a market-based economy [3]. Massive social change has occurred as a result of this transition [3]–[5]. The transition has promised improved living standards, and markedly increased choice in consumption, education, health, and employment for the Chinese population. It also presents many challenges, such as an imbalance between urban and rural development, rampant corruption, and a widening chasm between rich and poor [6], [7]. Studies suggest that people experiencing such conditions endure high stress [8], [9]. For example, one study showed that 64% of urban residents manifested moderate or high levels of stress and 22% suffered from mental disorders [9].

Stress now represents a major public health problem in China, with an estimated 173 million Chinese adults having a mental disorder [10]. Stress-related health problems have escalated. The World Health Organization (WHO) estimated that neuropsychiatric conditions and suicide together accounted for more than 20% of the total illness burden of China in 2004 [11]. By virtue of its large population, even a conservative estimate indicates that China has the largest number of reported suicides in the world. Globally, these deaths represent between 25% and 33% of all documented suicides. At least 600–800 Chinese kill themselves daily [12]. In recent decades, there has been a striking increase in Chinese alcohol consumption and associated problems [13], [14]. While national population-based studies of stress are rare in China, the need for them is great. Most extant stress studies have been confined to local subpopulations, and therefore provide no basis for estimating large-scale population-based stress levels [15]–[17]. The main impediment to generating such estimates is the large geographic and population size of China. Moreover, the country is culturally and socioeconomically diverse. Appropriate data on stress levels are nevertheless essential for informing health policy, planning prevention strategies, and designing and implementing appropriate and targeted interventions. To facilitate these purposes, our primary research aim was to estimate stress levels and secondary aim to identify sociodemographic correlates of stress.

## Methods

This study utilized a five stage cluster sampling technique. This sampling technique is “used in situations where the study population is very large and/or spread over a large geographic area” [18]. In Stage 1, six Chinese cities were selected according to geographic location: Hangzhou, Nanjing, Guangzhou, Taiyuan, Yinchuan, and Harbin. Capitals of their respective provinces, they were selected to ensure regional diversity. Harbin, with a population of 9.87 million, is located in the northeast and Taiyuan (3.46 million) is located in the north. Both are manufacturing cities. Yinchuan (1.62 million), located in the northwest, is characterized by agriculture, meat processing, and light industry. Nanjing (6.17 million), in the east, is dominated by science, education, and light industry. Hangzhou (6.72 million), in the southeast, features light industry and tourism. Guangzhou (7.74 million), located in the south, is characterized by light industry and commercial development. In terms of economic development, Harbin, Yinchuan and Taiyuan are at a low level, Nanjing is at a moderate level, and Hangzhou and Guangzhou are advanced [19]. Stage 2 comprised a random selection of residential districts in each city, excluding new building districts and subdistricts. In Stage 3, four ‘Jiedao’ (subdistrict neighborhoods) were then randomly selected within each residential district, and 16 building blocks were randomly selected from each ‘Jiedao’. In Stage 4, a family household registration (“hukou”) list was used to randomly sample households within the selected building blocks. Individuals aged 15 years and older, who had resided in their home for at least one year, were identified within each household. In the fifth and final stage, one of these eligible residents was then selected for interview based on birthdate closest to the date of contact.

A face-to-face interview was scheduled once an individual was identified and agreed to participate. All interviews were conducted in private by trained interviewers using a brief questionnaire. The same interview protocol was used across the six study cities in order to ensure homogeneity in interviewing and data collection. Data were collected between March and May 2008. The study was approved by the Ethics Committee at the Zhejiang University Medical Center, and informed written consent was obtained from all participants prior to the commencement of the study.

Possessing acceptable psychometric properties, measures adopted for this study have been used extensively in prior research on stress in Chinese populations [9], [19]. Stress, the dependent variable, reflected symptoms tapped by the Perceived Stress Scale, Chinese version (CPSS) [9], [20]–[21]. This scale comprised 14 items that addressed perceptions of stress during the month prior to the survey. Items were rated on a 5-point Likert-type scale and ranged from 0 (never) to 4 (very often). Item scores were summed to yield a total, with higher scores indicating higher perceived levels of stress [9], [20]. Following previous practice, severe stress was operationalised as a score > = 25 [9]. The internal reliability of the CPSS in this sample, measured by Cronbach's Alpha, was 0.85. Self-reported stress levels have been widely used to assess stress in many countries [22]–[25], and have been shown to be an appropriate indicator of mental health status [22]–[25].

Independent variables comprised a series of individual-level variables and one contextual-level variable. The individual-level variables covered sociodemographic characteristics: age (date of birth), educational attainment, marital status, occupation, and personal income. For the contextual variable, region, mainland China was divided into two broad geographic areas, north and south. Taiyuan, Yinchuan, and Harbin represented the north, and Hangzhou, Nanjing, and Guangzhou the south.

Data analysis was conducted in several stages using SAS version 6.12 and MLwiN Version 2.02 [26]. Standard algorithms were used to calculate stress scores. Means and 95% confidence intervals were calculated for the univariate description. Correlates of stress were evaluated using a multilevel variance component model [26], [27]. A nested hierarchical multilevel modeling technique possesses substantial advantages over a single-level regression procedure, when there is both a defined outcome measure and clear differentiation between ‘individual’ and ‘place’ or context [27]. By modeling random variation at both individual and regional levels, the study design could preclude ecological and atomistic fallacies, and thus distinguish the effects of individual and contextual variables upon stress. This approach permitted us to model the average association between stress and explanatory variables across the two regions.

We constructed two models. The first was the ‘null’ model, a two-level model of random intercepts. This base model was restricted to a constant in accounting for variation in stress scores across regions. In this base model, individual-level variables and the regional-level variable were entered as fixed main effects, given significant univariate associations with stress, to form the final model (Model 1). This enhanced model indicated how individual and regional variables in concert might explain inter-regional variation in stress levels. Model fitting was assessed by the likelihood of a change in -2 log. Significance of the random parameter variance estimates was assessed using the Wald joint X^2^ test statistic [26]. Our analysis was weighted by city population. Since differences in general characteristics were minimal within a given city [28], our weighting did not factor in district, subdistrict, apartment building block, or household.

## Results

We contacted a total of 5,333 individuals, whom we identified from our sampling list. Of this total, 4,981 agreed to interview, which yielded a survey response rate of 93%. Complete data were available on 4,735 participants (89% participation rate): 796 from Hangzhou, 791 from Nanjing, 911 from Yinchuan, 783 from Harbin, 695 from Guangzhou, and 758 from Taiyuan. Non-responders gave no reason for non-participation, and provided no other information.

The average stress score for the 4,735 respondents was 22.34(SD: 3.22; 95% C.I: 22.11–22.57). We found that 36.8% (95% CI: 33.5–40.2) were severely stressed, where severe stress was operationalized as an individual having a score >25 [9], [20]. Our univariate data indicated that resident age, education attainment, marital status, occupation, and income were all associated with stress (Table 1). Within specific sociodemographic groups, stress appeared lowest among the elderly, and highest among the educated, married, managers and clerks, and more affluent. Regionally, residents who lived in the south appeared less stressed than counterparts in the north.

**Table 1:** Individual characteristics and mean stress scores.

	N = 4735 (% of total sample)	Mean (95% CI)
**Individual level variables**		
***Age*** (in years)		P<0.01
<25	751 (15.9)	22.90 (22.68–23.12)
25–34	1259 (26.6)	22.62 (22.45–22.79)
35–44	964 (20.4)	23.02 (22.83–23.21)
45–54	796 (17.6)	22.94 (22.73–23.15)
55+	907 (19.6)	20.53 (20.29–20.78)
***Gender***		P>0.05
Male	2706 (57.1)	22.29 (22.17–22.42)
Female	2029 (42.9)	22.41 (22.27–22.55)
***Education***		P<0.01
Elementary school or lower	344 (7.3)	22.22 (21.85–22.59)
Junior high school	1033 (21.8)	22.23 (22.03–22.43)
High school	1289 (25.1)	23.28 (23.11–23.45)
Junior college	966 (20.4)	22.28 (22.08–22.48)
College or higher	1203 (25.4)	21.42 ( 21.24–21.60)
***Marital status***		P<0.05
Never married	1307 (27.6)	22.96 (22.79–23.13)
Married	3232 (68.3)	21.98 (21.87–22.09)
Other (divorced, widowed, separated)	196 (4.1)	23.64 (23.17–24.11)
***Ethnicity***		P>0.05
Han	4376 (92.4)	22.35 (22.04–22.66)
Other	359 (7.6)	22.71 (22.45–22.97)
***Occupation***		P<0.05
Managers and clerks	505 (10.7)	20.13 (19.86–20.39)
Professionals	549 (11.6)	22.44 (22.18–22.70)
Commercial and service workers	862 (18.2)	23.06 (22.86–23.26)
Students or army personnel	693 (14.6)	23.31 (23.08–23.54)
Operational workers	505 (10.7)	22.50 (22.27–22.74)
Unemployed	235 (5.0)	24.44 (24.03–24.85)
Retirees	764 (16.1)	20.75 (20.45–21.21)
Other	622 (13.1)	23.25 (23.00–23.50)
***Income/person/year(RMB)***		P<0.01
40,000+	756 (16.0)	20.44 (20.21–20.67)
<10,000	1144 (24.2)	23.40 (23.22–23.59)
10,000–19,999	1363 (28.8)	23.01 (22.84–23.18)
20,000–29,999	927 (19.6)	22.48 (22.28–22.68)
30,000–30,999	545 (11.5)	20.28 (20.02–20.53)
**Regional level variables**		
***Region***		P<0.01
South	2283	21.93 (21.81–22.00)
North	2452	22.66 (22.52–22.80)

Table 2 reports the results of the multilevel variance component model analyses of stress scores. In the null model, the random coefficient estimates indicated significant inter-regional variation in stress (P<0.01). Model 1 indicated that urban residents aged 35–44 years had higher stress levels and those aged 55 years and older lower stress levels than residents under age 25. All other occupational groups, except retirees and operations workers, manifested higher stress levels than their referent, managers and clerks. The highest educated residents exhibited lower stress levels than the least educated, and residents earning at least 20,000 RMB annually appeared less stressed than those earning less than 10,000 RMB. Finally, residents in the north had higher stress levels than those in the south, adjusting for individual-level sociodemographic characteristics.

**Table 2:** Results of multilevel variance component model analyses of stress scores.

	Null Model	Model 1
	Coefficient (95% CI)	Coefficient (95% CI)
**Fixed Effects**	22.562 (22.277–22.866)**	23.276 (21.576–24.976)**
***Individual level variables***		
***Age*** (in years)		
<25		Referent
25–34		0.701 (−0.522–1.924)
35–44		1.603 (0.594–2.232)**
45–54		0.416 (−0.648–1.480)
55+		−1.498 (<−0.609>–<−2.743>)**
***Occupation***		
Managers and clerks		Referent
Professionals		1.806 (1.230–2.384)**
Commercial and service workers		1.595 (0.537–2.653)**
Students or army		1.778 (0.596–2.960)**
Operational workers		0.569 (−0.584–1.714)
Unemployed		2.277 (0.879–3.675)**
Retirees		0.509 (−0.792–1.811)
Other		1.996 (0.889–3.103)**
***Education***		
Elementary school or lower		Referent
Junior high school		−0.791 (0.354–<−1.151>)
High school		−0.078 (0.072–<1.228>)
Junior college		−0.779 (0.407–<2.637>)
College or higher		−1.483 (<−0.238–<−2.728>)**
***Income/person/year(RMB)***		
<10,000		Referent
10,000–19,999		−0.0791 (0.621–<−0.778>)
20,000–29,999		−0.769 (<−0.013>–<−1.526>)**
30,000–30,999		−2.865 (<−1.895–<−3.835>)**
40,000+		−0.563 (−0.371–<−0.775>)**
***Regional level variables***		
***Region***		
South		Referent
North		0.619 (0.044–1.194)*
**Random Effects**		
**Level 1**	4.757 (2.385–7.129)**	3.635 (1.614–5.655)**
**Level 2**	9.646 (8.739–10.553)**	9.261 (4.494–14.028)**

*Significant at *p*≤.05.

**Significant at *p*≤.01.

## Discussion

Based on the results of this study, 36.8% (95% CI: 33.5–40.2) of urban residents were severely stressed. Our finding that more than one-third of urban residents are severely stressed likely harbors serious ramifications for China. Numerous studies have shown escalation of stress-related health problems, and that stress has emerged as a serious public health issue for the urban population [6]–[14], [19]. Since 1978, massive social change occurred in China with profound consequences for the population [3]–[5]. More people are facing increased rates of unemployment, higher living costs, increased inequality, lack of resources, increased pressure to produce, and general uncertainty about their future [6], [7]. This constellation of pressures makes them highly prone to stress-related problems [8], [9]. Many studies have reported that severe and persistent stress may induce poor health, and generally adversely influence quality of life [1]–[2], [21]–[25]. Stress now represents a major public health problem in China, with an estimated 173 million Chinese adults having a mental disorder [10]. China also has the largest number of reported suicides in the world [12]. In recent decades, there has been a striking increase in alcohol consumption and related problems in China, which may also reflect the adverse impact of high stress levels [14].

In this study, significant differences (p<.01) were found between the reported stress levels of those living in northern China versus southern China. The regional differences reported here, however, may reflect substantial variation in both socioeconomic development and cultural norms between the north and south [20], [29]–[31]. Culturally, the north has been influenced by a nomadic culture and the south by an agrarian one [20], [29]. Generally, residents in the north regard each event as determined by nature. It can be extremely frustrating for them to observe the socioeconomic transitions taking place; especially when they are not beneficiaries. This may exacerbate their stress. Furthermore, economic development has occurred faster in the south, and due to their agrarian lifestyle, southern residents may be more content and less frustrated with any economic imbalances that exist. Subsequent analysis showed that while variation in stress scores occurred regionally (33.5%), individual-level variation was greater (66.5%); that is, stressors appear more individual than regional.

A number of sociodemographic variables, which we incorporated in Model 1, were associated with stress. Urban residents aged 35–44 years were more stressed than counterparts aged 55 years and older. This is probably due to the fact that members of the 35–44 age group are simultaneously facing the pressures of establishing careers and raising families. Members of the 55 and older age group have already established their careers and seen their families mature, and are nearing retirement age. The middle-aged are at a stressful and challenging time in their life cycle. Occupationally, they are likely to be more encumbered by responsibility than younger counterparts. Domestically, they can face formidable challenges from their adjacent generations – often needing both to care for elderly parents and meet the needs of children who are older teens or younger adults. People of different ages may well face unique stressors that will require creative, unique and individualized approaches for effective stress-management.

Confirming prior findings, education and income were both associated with stress [9], [20]. People with higher educational attainment are able to obtain and use more resources than those with less education. Moreover, as part of their educational process, they may have learned better coping skills for confronting life challenges [15], [31]–[32]. This study also confirmed findings that income is a crucial social resource, and that urban residents of higher socioeconomic status experience less stress than others. Higher economic attainment means better educational opportunities and social networks, more personal freedom, and healthier and safer work environments [20], [28], [33]–[34].

Stress also varied by occupation. Professionals, commercial and service workers, the unemployed, army personnel and students were more stressed than managers and clerks. This finding also confirmed prior findings [9], [19]. We suspect that occupational variation in stress primarily reflects variable socioeconomic status. For example, the unemployed showed high levels of stress while managers and clerks manifested relatively little stress. The unemployed are in a very precarious socioeconomic situation, while managers and clerks have relatively stable socioeconomic status [34]. Job function likely also determines stress levels. For example, professionals, commercial and service workers have high-stress jobs with great social competition [20], [34]. On the other hand, clerks and managers have relatively less social competition and consequently less stress.

We compared our results, specifically regarding the stress levels of urban males (mean = 22.29, 95% CI: 22.17–22.42), with those of a study of stress levels among rural males (mean = 24.8, 95% CI: 24.6–25.0) [20]. The rural males have significantly higher reported stress levels than the urban males even though both groups exhibit high levels. This difference may reflect the fact that China has experienced rapid socioeconomic development in urban areas, with a much slower transition occurring in rural areas [35]. Thus, while overall living standards have risen across China, there has been a lag in rural farmers receiving the benefits. Per capita annual income for rural residents is only 4,761 renminbi (RMB), as compared to 15,781 RMB for urban residents. Many rural Chinese dwell in cramped, dank, and dilapidated mud-clay housing [36]. They are usually confined to difficult agricultural jobs, and cannot access important benefits available to urban residents, such as social welfare and free health care. Due to poverty and a paucity of social welfare programs, minor illness or injury has to be endured, and serious illness may well produce death or permanent disability. Pressure and fatigue are life constants for rural residents. As a consequence of their existence, they are likely at excess risk for stress and mental health disorders [20], [36], [37]–[38].

Based on the results of this and prior studies, prevention must target both urban and rural residents at high risk of severe stress, especially those of low socioeconomic status. The poor and uneducated lack the resources and skills to seek assistance for their stress-related mental health issues. As a first step, the Chinese government needs to review its policies and implement new initiatives to reduce income inequality and guarantee benefits to the large disadvantaged element of society. If the gap between those in high versus low socioeconomic groups can be narrowed, stress and mental health issues will be reduced.

Beyond reducing social and economic barriers, more needs to be done to assist those facing high stress and associated mental health problems. Both government and local health authorities need to develop programs to prevent and treat stress-related mental health disorders, and to reduce the high prevalence of severe stress now evident in China. A multifaceted approach to prevention and treatment should be incorporated into community healthcare programs. For example, local health authorities should offer education in stress management and concurrently offer mental health treatment as needed. A health education curriculum should be provided in all schools and universities to help young people recognize the signs of stress, and to teach them skills to manage their stress. Special community-based clinics should be established to provide high-risk individuals with no or low cost psychological counseling on an as-needed basis. Support groups should be established in urban community centers to provide a forum where residents can express mental health concerns, talk with others having similar concerns, and learn stress-management skills. Worksite programs should be established to help employees manage their stress at work and at home. Employers should also be incentivized to adopt policies that will reduce the stress of their employees. This would be a win-win for employers and employees. Employees would receive stress-management skills and assistance in coping with stress, and employers would have healthier and hence more able employees.

This study has several limitations. First, the study population was drawn from several large capital cities, and thus they may not represent all cities in China. However, in order to achieve a high degree of large city representation, in our selection we were careful to utilize criteria adopted in previous studies, and to select cities from different geographic regions with variable levels of economic development. Our survey was large-scale and study results are likely a strong indicator of the extent and degree of stress-related issues within the Chinese urban population. Secondly, this study used a cross-sectional design, which thus precluded causal inference. A third limitation is that stress was based on self-report, and therefore results may underestimate or overestimate true stress levels. We cannot determine the extent of self-reporting bias. Our instrument for measuring stress, the Perceived Stress Scale, Chinese version (CPSS), possesses acceptable reliability and validity that is empirically based [9], [20]. There is a crucial need to collect longitudinal data on stress in urban communities, with multiple time points for the purpose of conducting surveillance, prevention, and evaluation. In addition, future studies need to focus on how community-level health programs, government regulations, and targeted stress-management campaigns can impact stress levels, and ultimately improve the mental health and health status of the Chinese population.

This research contributes to the general stress literature by documenting high stress levels among urban residents in selected China cities. This study indicates that stress levels are variably associated with social class, income, education, geographic location, type of occupation, and age. There is an imperative for government and local health agencies to collaborate in developing and implementing a mix of policies and programs to markedly reduce severe stress among the Chinese urban populace. These actions must address primary, secondary and tertiary levels of prevention, with all policies and programs being appropriately evaluated in due course.

## References

[1] Field TM, McCabe PM (1985). Stress and coping.

[2] Charney DS, Manji HK (2004). Life stress, genes, and depression: multiple pathways lead to increased risk and new opportunities for intervention.. Sci STKE.

[3] Hao C (2006). Development of financial intermediation and economic growth: The Chinese experience.. China Economic Review.

[4] Chen BZ, Feng Y (2000). Determinants of economic growth in China: Private enterprise, education, and openness.. China Economic Review.

[5] Lin JY (1992). Rural reforms and agricultural growth in China.. The American Economic Review.

[6] Zhou XG (2000). Economic transformation and income inequality in urban China: Evidence from panel data.. American Journal of Sociology.

[7] Meng X (2004). Economic restructuring and income inequality in urban China.. Review of Income and Wealth.

[8] Phillips MR, Liu HQ, Zhang YP (1999). Suicide and social change in China.. Culture Medicine and Psychiatry.

[9] Yang TZ, Huang HT (2003). An epidemiological study on stress among urban residents in social transition period.. Chinese Epidemiology.

[10] Phillips MR, Zhang JX, Shi QC, Song ZQ, Ding ZJ (2009). Prevalence, treatment, and associated disability of mental disorders in four provinces in China during 2001–05: an epidemiological survey.. Lancet.

[11] World Health Organization (2009). Department of Measurement and Health Information. Death and DALY estimates for 2004 by cause for WHO Member States.. http://www.who.int/healthinfo/bod/en/index.html.

[12] Lee S, Kleinman A, Perry EJ, Seldon M (2000). Suicide as resistance in Chinese society.. Chinese society: Change, conflict and resistance.

[13] Cochrane J, Chen HH, Conigrave KM, Hao W (2003). Alcohol use in China.. Alcohol and Alcoholism.

[14] Hao W, Su ZH, Liu BL, Zhang K, Yang HQ (2004). Drinking and drinking patterns and health status in the general population of five areas of China.. Alcohol and Alcoholism.

[15] Tang CSK, Au WT, Schwarzer R, Schmitz G (2001). Mental health outcomes of job stress among Chinese teachers: role of stress resource factors and burnout.. Journal of Organizational Behavior.

[16] Chen WQ, Yu IT, Wong TW (2005). Impact of occupational stress and other psychosocial factors on musculoskeletal pain among Chinese offshore oil installation workers.. Occup Environ Med.

[17] Liu XC, Kurita H, Uchiyama M, Okawa W, Liu LQ (2000). Life events, locus of control, and behavioral problems among Chinese adolescents.. Journal of Clinical Psychology.

[18] Cottrell RR, McKenzie JF (2011). Health Promotion & Education Research Methods.

[19] Department of Comprehensive Statistics of National Bureau of Statistics (2009). China statistics China yearbook for regional economy, 2008.

[20] Yang T, Rockett IR, Yang X, Xu X (2009). Patterns and correlates of stress among rural Chinese males: a four-region study.. Public Health.

[21] Cohen S, Kamarck T, Mermelstein R (1983). A global measure of perceived stress.. J Health Soc Behav.

[22] Edmondson KA, Lawler KA, Jobe RL, Younger JW, Piferi RL (2005). Spirituality predicts health and cardiovascular responses to stress in young adult women.. Journal of Religion and Health.

[23] Gary TL, Stark SA, Gary TL, LaVeist TA (2007). Neighborhood characteristics and mental health among African Americans and whites living in a racially integrated urban community.. Health & Place.

[24] Farley T, Galves AL, Dickinson LM, Perez MDJ (2005). Stress, coping, and health: a comparison of Mexican immigrants, Mexican-Americans, and non-Hispanic whites.. Journal of Immigrant Health.

[25] Deckro GR, Ballinger KM, Hoyt M, Wilcher M, Dusek J (2002). The evaluation of a mind/body intervention to reduce psychological distress and perceived stress in college students. http://dx.doi.org/10.1080/07448480209603446.

[26] Rashash J, Steele F, Browne WJ, Goldstein H (2001). A user's guide to MLinN Version 2.10.Centre for Multilevel Modeling.

[27] Goldstein H (1995). Multilevel statistical models (second edit).

[28] Fone DL, Dunstan F (2006). Mental health, places and people: a multilevel analysis of economic inactivity and social deprivation.. Health & Place.

[29] National Bureau of Statistics of China. Results of the sixth national census.. http://www.china.com.cn/policy/txt/2011-04/28/content_22459130.

[30] Tseng WS, Wu DYH, Wu D (1985). Chinese Culture and Mental Health.

[31] Ritter C, Hobfoll SE, Lavin J, Cameron RP, Hulsizer MR (2000). Stress, psychosocial resources, and depressive symptomatology during pregnancy in low-income, inner-city women.. Health Psychol.

[32] Paul K, Boutain D, Agnew K, Thomas J, Hitti J (2008). The relationship between racial identity, income, stress and C-reactive protein among parous women: implications for preterm birth disparity research.. J Natl Med Assoc.

[33] Cacioppo JT, Hughes ME, Waite LJ, Hawkley LC, Thisted RA (2006). Loneliness as a specific risk factor for depressive symptoms: Cross-sectional and longitudinal analyses.. Psychol Aging.

[34] Liu X (2002). Ten social stratums in China society.. Xu Xi He Shi Jian.

[35] Guo J (2001). Features and problems in increment of farmers' incomes in current stage in China with some countermeasures.. Chinese Rural Economy.

[36] Review of China economic development.. http://www.china.com.cn/aboutchina/txt/2009-01/22/content_17169729htm.

[37] Zhang S (2003). Agriculture farmer and countryside problems in mainland China.

[38] Pu Y, Ji R (2008). Health education need of left behind women.. Chinese Journal of Health Education.

